# A novel approach for heart disease prediction using strength scores with significant predictors

**DOI:** 10.1186/s12911-021-01527-5

**Published:** 2021-06-21

**Authors:** Armin Yazdani, Kasturi Dewi Varathan, Yin Kia Chiam, Asad Waqar Malik, Wan Azman Wan Ahmad

**Affiliations:** 1 Department of Software Engineering, Faculty of Computer Science and Information Technology, Universiti Malaya, Kuala Lumpur, Malaysia; 2 Department of Information Systems, Faculty of Computer Science and Information Technology, Universiti Malaya, Kuala Lumpur, Malaysia; 3 Department of Computing, National University of Sciences and Technology (NUST), Islamabad, Pakistan; 4 Department of Medicine, Faculty of Medicine, Universiti Malaya, Kuala Lumpur, Malaysia

**Keywords:** Weighted associative rule mining, Heart disease prediction, Cardiovascular disease, Weighted scores

## Abstract

**Background:**

Cardiovascular disease is the leading cause of death in many countries. Physicians often diagnose cardiovascular disease based on current clinical tests and previous experience of diagnosing patients with similar symptoms. Patients who suffer from heart disease require quick diagnosis, early treatment and constant observations. To address their needs, many data mining approaches have been used in the past in diagnosing and predicting heart diseases. Previous research was also focused on identifying the significant contributing features to heart disease prediction, however, less importance was given to identifying the strength of these features.

**Method:**

This paper is motivated by the gap in the literature, thus proposes an algorithm that measures the strength of the significant features that contribute to heart disease prediction. The study is aimed at predicting heart disease based on the scores of significant features using Weighted Associative Rule Mining.

**Results:**

A set of important feature scores and rules were identified in diagnosing heart disease and cardiologists were consulted to confirm the validity of these rules. The experiments performed on the UCI open dataset, widely used for heart disease research yielded the highest confidence score of 98% in predicting heart disease.

**Conclusion:**

This study managed to provide a significant contribution in computing the strength scores with significant predictors in heart disease prediction. From the evaluation results, we obtained important rules and achieved highest confidence score by utilizing the computed strength scores of significant predictors on Weighted Associative Rule Mining in predicting heart disease.

## Introduction

Cardiovascular disease (CVD) is one of the most life-threatening diseases in the world. The World Health Organization (WHO) as well as the Global Burden of Disease (GBD) study reported cardiovascular disease as the main cause of death around the globe annually [40, 56].  WHO revealed that CVD is expected to affect almost 23.6 million people by the year 2030. In some industrialized countries such as the United States of America, the rate is about 1 in 4 deaths [34]. The Middle East and North Africa (MENA) region has an even higher percentage, which is 39.2% of the mortality rate [20]. Hence, early and accurate diagnosis and the provision of appropriate treatments are keys to reducing the amount of death causing cardiovascular diseases. Availability of such services is essential for those who have a high risk of developing heart disease [29].

There are many features that contribute to heart disease prediction. Researchers in the past were more focused on identifying significant features to be used in their heart disease prediction models [8]. Less importance was given to determining the relationships between these features and to identifying their level of priority [32, 32] within the prediction model. To address the issues which hinder early and accurate diagnosis, many data mining related studies were previously conducted [9, 16, 28].

Weighted Association Rule Mining (WARM) is one of the data mining techniques used to discover the relationships between features and to determine mining rules that lead to certain predictions [22]. The weight that is used in this mining technique provides users with a convenient way to indicate the importance of the features that contributes to heart disease and helps obtain more accurate rules [4]. In many prediction models, different features have different importance. Hence, different weights are assigned to different features based on their predicting capabilities [48]. The failure in determining the weight indicates the failure in determining the importance of the features.

Past research had used Weighted Associative Rule Mining (WARM) in heart disease prediction [18, 31, 46, 48, 50]. However, the prediction model reported in these studies still demands further exploration in terms of the number of features used, the strength of these features and the evaluation of scores obtained. In this research, we proposed an algorithm to compute the weight of each feature that contributes to heart disease prediction. We have experimented on all features as well as selected significant features using WARM. The results obtained showed that the significant features outperformed all features with the highest confidence score of 98% in predicting heart disease. To the best of our knowledge, this study is the first that used strength scores of significant predictors in WARM.

The rest of the paper is organized as follows: Sect. 2 presents the background of the study followed by Sect. 3 on research objectives. Section 4 presents the methodology and Sect. 5 displays the results obtained by this research. Section 6 includes the discussions and Sect. 7 benchmarks this research against previous studies. Finally, Sect. 8 concludes the research with a summary of the findings and future work.

## Related works

CVDs are disorders of the heart and blood vessels and include coronary heart disease, cerebrovascular disease and other conditions. Heart attacks and strokes are the main causes of mortality in cardiovascular disease in which the rate nears one out of three [6]. With the high rate of mortality, diagnosis and prevention measures need to be performed effectively and efficiently. Many data mining techniques have been used to help address these issues (Amin et al. [8]). Most of the past research looked into identifying features that contribute to better heart prediction accuracy [9]. However, very little researches looked into the relationships that exist between these features. The relationship between each feature that contributes to heart disease prediction can be obtained by using the Associative Rule Mining (ARM) technique [11]. The ARM technique is popular in transactional and relational datasets. The hidden knowledge in large datasets such as business transactions developed the interest of many business owners to understand the patterns that can help them to improve their business decisions (Agarwal and Mithal [1]). For instance, discovering the frequently bought items by customers in market basket analysis. This analysis looks at the various items found in customers’ shopping cart and identifies the associations between them. A good example would be if customers were looking to purchase milk, they were likely to purchase bread on the same trip to the supermarket. This approach is also widely used in the healthcare industry specifically in privacy preservation of healthcare data [15], predicting cancer associated protein interactions [12], predicting obstructive sleep apnea [43] and predicting co-diseases in Thyroid patients [23].

ARM is also used in heart disease prediction. Table 1 shows the studies that used ARM in heart disease prediction. Akbaş et al. [3], Shuriyaa and Rajendranb [42], Srinivas et al. [49], Khare and Gupta [24] and Lakshmi and Reddy [27] have used ARM on UCI dataset. Some of the studies listed in Table 1 used private datasets from hospitals and heart centres. Although the scores that were obtained from these datasets are high (99% by Sonet et al. [45]), 100% by Thanigaivel and Kumar [52], the studies have a limitation in terms of reproduction, as the datasets are not open for access. Akbaş et al. [3] on the other hand obtained a score of 97.8% in confidence using the UCI dataset. However, the confidence score obtained predicted people with no risk of heart disease.

**Table 1:** Studies on Heart Disease Prediction using ARM

Authors	Technique	No of Features Used	Evaluation Metric	Score	Dataset
Akbaş et al. [3]	Associative Rule Mining	13	Confidence	97.8 (Predicting no heart disease)	UCI
Vasanthanageswari and Vanitha [54]	Associative Rule Mining	16	NA	NA	Congenital Heart Defect Dataset
Shuriyaa and Rajendranb [42]	Associative Rule Mining + ANFIS	13	Accuracy	93.2	UCI
Sonet et al. [45]	Associative Rule Mining	13	Confidence	99	National Institute of Cardiovascular Disease, Dhaka, Bangladesh
Thanigaivel and Kumar [52]	Associative Rule Mining	25	Confidence	100	Hospital (name of the hospital not mentioned)
Srinivas et al. [49]	Associative Rule Mining and MLP	13	Accuracy	84.9	UCI
Khare and Gupta [24]	Associative Rule Mining	13	Confidence	94	UCI
Lakshmi and Reddy [27]	Associative Rule Mining	13	Accuracy	96.6	UCI
Said et al. [41]	Associative Rule Mining	13	Confidence	91	UCI
Nahar et al. [36]	Associative Rule Mining	13	Confidence	96	UCI

Weighted Associative Rule Mining (WARM) is an extension of ARM, in which weights are assigned to differentiate the importance of the features mined. Let T be the training dataset in which contains T = {r_1_, r_2_, r_3_… r_i_} with a set of weight associated with each {attribute, attribute value} pair. Every ith record r_i_ is a set of value and weight w_i_ attached to each feature of r_i_ tuple / record. In a weighted framework, each record is a set of triple {a_i_, v_i_, w_i_} where feature a_i_ has a value of v_i_ and weight of w_i_ where 0 < w_j_ <  = 1.

Assigning a correct weight to each feature is a hard task. In various fields of studies, there are different ways of calculating the weights of features. For instance, according to Malarvizhi and Sathiyabhama [30] in web mining, visitor page dwelling time is a way of calculating weightage. WARM is widely used in research on shopping basket scenarios and in predicting customers’ behaviour. Chengis et al. [10] investigated on assigning weight before and after ARM. WARM was also used in predicting disease comorbidities using clinical as well as molecular data (Lakshmi and Vadivu 26). This technique is also used in predicting breast cancer [5]. Recent research by Park and Lim [39] used this technique to reduce design failures of pre-alarming systems in the shipbuilding industry.

However, not many researchers focused on applying WARM to cardiovascular disease. Table 2 shows studies on heart disease prediction using WARM. However, the weight of features was not precisely calculated (Jabbar et al. [21], Sundar et al. [50], Soni and Vyas [48]). Soni et al. [47] proposed a new framework, which was an associative classifier that used WARM. Different weights were assigned to different attributes based on their predicting capability. Their theoretical model yielded a confidence score of 79.5%. Soni and Vyas [48] also applied WARM and the confidence level they achieved was was 79.5%. Their research assigned weights based on age range, smoking habits, hypertension and BMI range. On the other hand, Soni et al. [46] assigned weights to each of the attributes based on the advice obtained from the medical experts. They presented an intelligent and effective heart attack prediction system using a weighted associative classifier by achieving a maximum score of 80% confidence. Meanwhile, Sundar et al. [50] developed a system using two data mining techniques, which are Naïve Bayes and WARM. Their experiments showed that WARM achieved a score of 84% on confidence score, outperforming Naïve Bayes, which obtained only 78%. Chauhan et al. [11] also used WARM in predicting heart disease. They obtained an accuracy score of 60.4%. Kharya et al. [25] used Weighted Bayesian Association Rule Mining Algorithm, which combines WARM with heart disease dataset. However, they failed to indicate the results obtained in their study. Ibrahim and Sivabalakrishnan [19] have used Random Walker Memetic algorithm-based WARM for predicting coronary disease. They obtained an accuracy of 95% using the UCI heart disease dataset.

**Table 2:** Studies on Heart Disease Prediction using WARM

Authors	Technique	No of Features Used	Evaluation Metric	Score	Dataset
Ibrahim and Sivabalakrishnan [19]	Random Walk Memetic Algo with WARM	13	Precision	92%	UCI
Ibrahim and Sivabalakrishnan [18]	WARM	13	Confidence	67%	UCI
Kharya et al. [25]	WARM with Bayesian Belief Network	4	NA	NA	NA
Chauhan et al.[11]	WARM	13	Accuracy	60.4%	UCI
Sundar et al. [50]	WARM	13	Confidence	84%	UCI
Soni et al. [46]	WARM	13	Confidence	80%	UCI
Soni and Vyas [48]	WARM	13	Confidence	79.5%	UCI

Despite having research that is based on WARM in predicting heart disease, none of them was focused on identifying the important features to be used in heart disease prediction which would contribute to better prediction performance. The weight of each feature plays an equally important role in deciding which feature has the highest impact (strength) in predicting heart disease. The right weight of the significant features identified will yield an effective prediction model. Thus, this research is focused on identifying the weight of significant features and utilizing the generated score in predicting heart disease.

## Research objectives

The main objectives of this research are as follows:

To compute the weight of significant features in heart disease prediction.

To predict heart disease using the computed weight of significant features (using WARM).To evaluate the performance of WARM in predicting heart disease.

## Proposed methodology

**Fig. 1:**
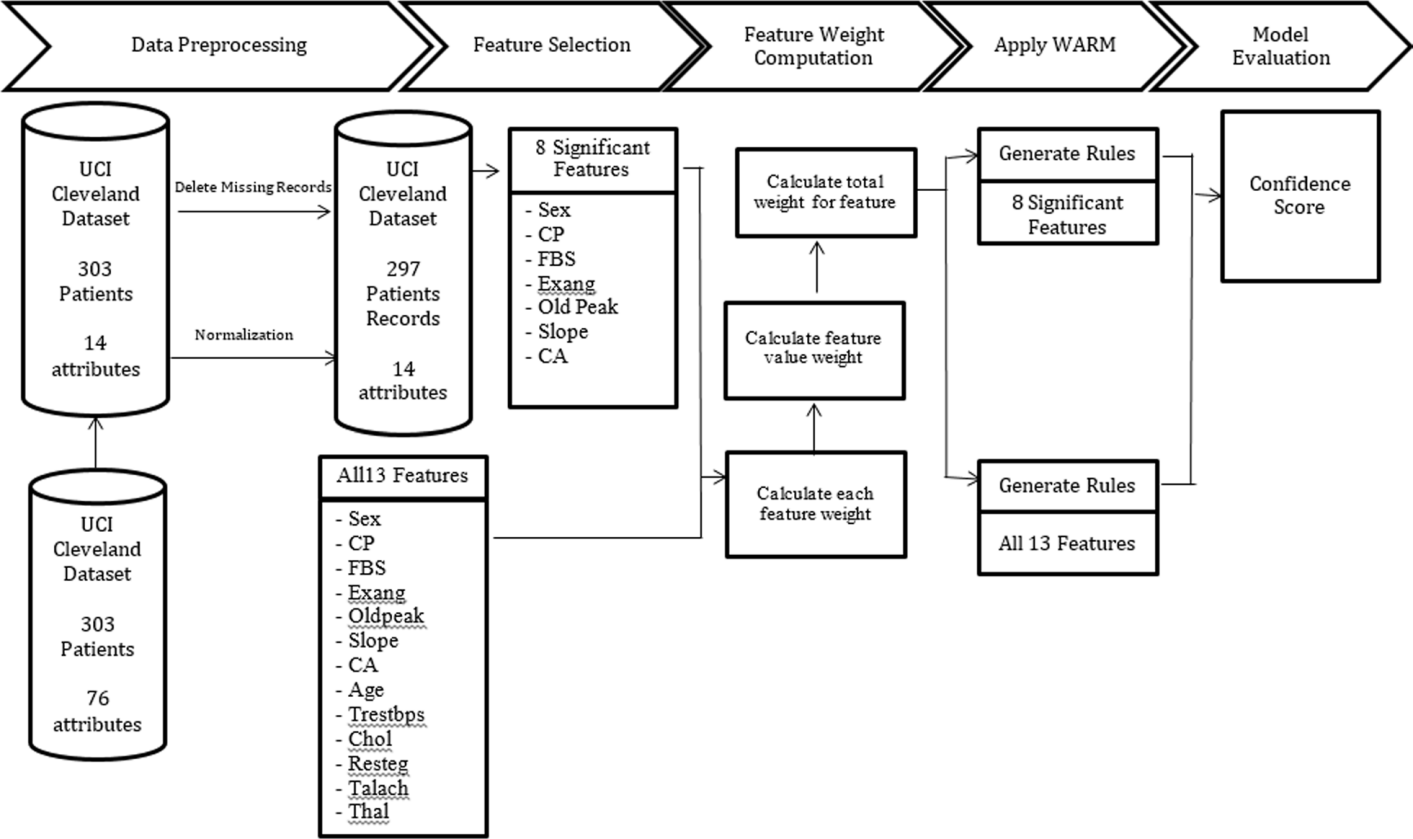
Methodology

This section describe in detail the methodology used as shown in Fig. 1. It contains 5 main stages which are data pre-processing, feature selection, feature weight computation, apply WARM and model evaluation.

### Dataset

This research uses the heart disease dataset that is obtained from UCI Machine Learning Repository [13]. UCI Machine Learning Repository is one of the largest available datasets, having over 417 various datasets. The Cleveland dataset from UCI Machine Learning Repository is one of the datasets on heart disease, which is widely used by researchers to date (Amin et al. [8]). This research will also use this dataset of which contains 303 rows. The dataset contains 76 features in which 14 attributes including class label are used. The 14 features together with their descriptions and data types are shown in Table 3.

**Table 3:** Features description

No	Features	Description	Data Type
1	Age	Age in year	Numeric
2	Sex	Gender	Nominal
3	CP	Chest pain type	Nominal
4	Trestbps	Resting blood pressure	Numeric
5	Chol	Serum cholesterol	Numeric
6	Fbs	Fasting blood sugar	Nominal
7	Resteg	Resting electrographic results	Nominal
8	Talach	Maximum heart rate achieved	Numeric
9	Exang	Exercise induce angina	Nominal
10	Oldpeak	ST depression induced by exercise relative to rest	Numeric
11	Slope	The slope of the peak exercise ST segment	Nominal
12	CA	Number of major vessels coloured by fluoroscopy	Numeric
13	Thal	Thallium heart scan	Nominal
14	Goal	Diagnosis of heart disease	Nominal

### Experimental Setup

In this research, Weka 3.8 was used to conduct the experiments. The retrieved Cleveland dataset went through a pre-processing phase. The significant features were retrieved from a total of 14 factors from the Cleveland dataset (Amin [7]). Further, the weight of each significant feature was computed and assigned back to them accordingly. WARM was applied to the heart disease dataset to generate rules. Finally, evaluation was performed to obtain the confidence score of the best rules generated using WARM based on significant features. The detailed explanation of each process is explained in the following sections.

### Data Pre-Processing

In the data pre-processing phase, all missing records were deleted from the dataset, which consists of 6 instances. Based on Table 3, there are 13 normal attributes(age’, ‘sex’, ‘cp’, ‘trestbps’, ‘chol’, ‘fbs’, ‘restecg’, ‘thalach’, ‘exang’, ‘oldpeack’, ‘slope’, ‘ca’, ‘thal’) and 1 class label(‘goal’), which refers to the criticality level of heart disease in patients. It ranged from 0–4, in which 0 refers to’No Heart Disease’ and the other values indicates the presence of heart disease at different criticality levels. Since this research aims at predicting the presence of heart disease and not its criticality levels, the range from 1 to 4 is thus normalized to 1, which indicates the presence of heart disease, and 0 to represent the absence of heart disease. Data normalization is also performed as a part of the data transformation process that involved mounting data into nominal data. This is required, as WARM utilizes nominal data only. All the ranges formed for each features are indicated in Table 4.

**Table 4:** Ranges formed for features

Age	< = 40: lessThanForty 41–64: betweenAge > = 65: greaterThanSixtyFour
Sex	1: Male 0: Female
CP	1: typicalAngina 2: atypicalAngina 3: nonAnginalPain 4: asymptomatic
Trestbps	90–120: normal 120–140: unusual 140–160: high > 160: very high
Cholesterol (chol)	110–200: normal 200–240: borderline_high 240–250: high > 250: very high
Fbs	True False
Restecg	0: normal 1: STTWaveAbnormality 2: showingProbable
Thalach	60–100: Normal > 100: Tachycardia
Exang	Yes No
Oldpeak	Zero greaterThanZero
Slope	1: Upsloping 2: Flat 3: Downsloping
CA	Zero One Two Three
Thal	3: Normal 6: Fixed 7: Reversible
Output	0: No Heart Disease 1: Heart Disease

*Source: *Khare et al. [24]

### Feature Selection

Features were selected based on experiments conducted by Amin et al. [8] since they had used the same dataset (UCI). They performed a set of experiments that dealt with 8100 combinations of features with 7 different classification models (K-NN, Decision Tree, Naïve Bayes, Logistic Regression, Neural Network and Vote) to identify significant features. Table 5 shows the features obtained from the highest performance of each classification models. The highlighted columns indicate the features which appeared more than 10 times and thus were selected as significant features. The selected 8 features are sex, CP, Fbs, Exang, Oldpeak, Slope, CA, and Thal.

**Table 5:** Selecting significant features from the result of the highest performance

	Age	Sex	CP	Trestbps	Chol	Fbs	Restecg	Thalach	Exang	Oldpeak	Slope	CA	Thal
Occurrence in Highest Accuracy	2	7	7	1	2	5	4	3	4	6	4	7	5
Occurrence in Highest F-Measure	2	7	7	1	2	5	4	3	4	6	4	7	5
Occurrence in Highest Precision	0	6	4	2	1	2	2	2	4	2	4	5	4
Total Occurence	4	20	18	4	5	12	10	8	12	14	12	19	14

*Source*: Amin et al. [8]

### Feature weight computation

This section explains how the weight of the features was calculated. The fundamental of WARM states that different features in a dataset have different importance in predicting heart disease. The weight of each feature ranges from 0 to 1. Thus, a weight that is closer to 1 indicates a more significant feature. On the other hand, a weight that is closer to 0 is the least significant in heart disease prediction.

#### Calculate feature weight

The first step was to calculate the individual feature weights. Let R be the set of features R = {n_0_, n_1_, n_2_… n_i_} and (n > 0). In this experiment, the total number of features is 13 and after feature selection, it is reduced to 8 (Sex, CP, Fbs, Exang, Oldpeak, Slope, CA, and Thal). W (n) is the weight of each feature (W is the weight of each feature to be calculated and n represents a feature),

1 $$W\left( n \right) = \frac{{\text{n}}}{{\mathop \sum \nolimits_{{{\text{n}}_{0} ,\;{\text{n}} \in {\text{R}}}}^{{\text{n}}} {\text{n}}_{0} + {\text{n}}_{1} + \cdots + {\text{n}}_{i} }}$$

For example, the value of sex as displayed in Table 5 is’20’ and the sum of all the features will be’121’. The total value of significant features (Sex, CP, Fbs, Exang, Oldpeak, Slope, CA, andThal) is calculated as (20 + 18 + 12 + 12 + 14 + 12 + 19 + 14 = 121). Thus, to calculate the weight of ‘sex’ (weight of features, WOF):

$${\text{WOF}} = {\text{W}}\left( {20} \right) = 20/121 = 0.17$$

Table 6 displays the calculated weights for each of the significant features. All weights were computed accordingly. From the distribution of the weights, CA has the greatest strength followed by Sex, CP, Oldpeak and Thal, Fbs, Exang and Slope has the similar weight of 0.09 each.

**Table 6:** Weight of the significant features

Sex	0.17
CP	0.15
Fbs	0.09
Exang	0.09
Oldpeak	0.12
Slope	0.09
CA	0.18
Thal	0.11

#### Calculate feature value weight

This section explains how feature values are computed. Feature values represent all the values that a feature contains. For instance, feature values for sex are male and female. Let A be the number of each feature value contained in the dataset and (A ∪ B) be the total number of records.

Table 7 shows the total sub value of each feature based on the UCI dataset. Male value is represented by 203 records and female by 94 records which gives a total of 297 records from the UCI dataset. To calculate the value of each feature weight, let A be the selected value and B be the rest of the features value,

2 $${\text{W}}_{{({\text{value}} = {\text{A}})}} = \frac{A}{A \cup B}$$

$$\begin{aligned} & {\mathbf{Gender}}\;{\mathbf{male}}\;{\mathbf{value}}:\;{\text{W}}_{(206)} = \, 203/297 = 0.68 \\ & {\mathbf{Gender}}\;{\mathbf{female}}\;{\mathbf{value}}:\;{\text{W}}_{(97)} = 97/297 = 0.32 \\ \end{aligned}$$

**Table 7:** Identify total sub value of each feature

Total	Male	Female
297	203	94

**Fig. 2:**
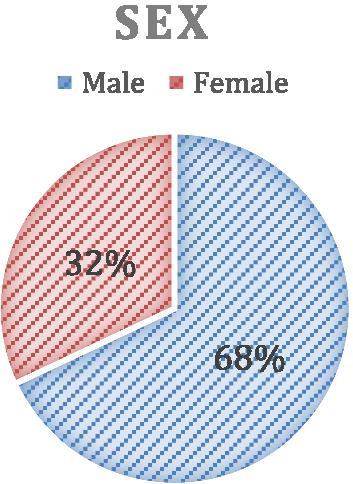
Comparison on the percentage of male and female in Cleveland heart disease dataset

Figure 2 shows the comparison of the percentage of males and females in the Cleveland heart disease dataset.

#### Calculate total weight for feature

This section explains how the total weight for features is computed. The feature weight (W (n)) and feature value weight (W (value)) gives the total weight (W (t)) for the feature. The computation is shown below.

3 $${\text{W}}\left( {\text{t}} \right) = {\text{W}}\left( {\text{n}} \right)*{\text{W}}\left( {{\text{value}}} \right)$$

Example of calculating the total weight of feature W (t):

$$\begin{aligned} & {\mathbf{Male}}:\;{\text{W}}\;\left( {{\text{total}}\;{\text{Male}}} \right) = 0.14*0.68 = 0.0952 \\ & {\mathbf{Female}}:\;{\text{W}}\;\left( {{\text{total}}\;{\text{Female}}} \right) = 0.14*0.32 = 0.0448 \\ \end{aligned}$$

### Algorithm

This section detailed out the algorithm to obtain the weighted score of each feature in predicting heart disease. The algorithm is stated as follows:
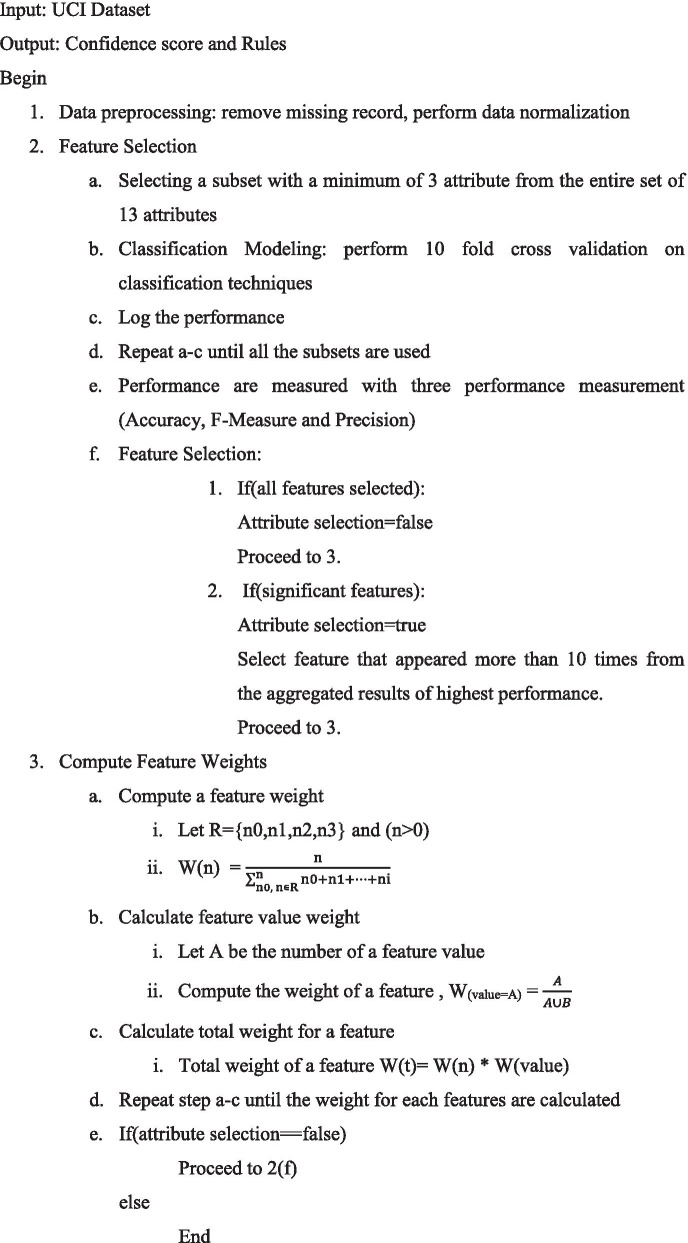


### Apply WARM

Not all features in the heart disease dataset have the same level of significance in predicting the risk of heart disease. Thus, different weights based on their prediction capability are assigned. These values are then imported into Weka 3.8 to experiment with WARM using Apriori Algorithm.

#### Apriori algorithm

The Apriori algorithm is a well-known approach in WARM. Apriori was first proposed by Agrawal and Srikant [2]. The algorithm starts with a dataset including transactions that wants to construct frequent item sets, having at least a user-specified threshold. In the algorithmic process of Apriori, an item set X of length k is frequent if and only if every subset of X, having length k—1, is also frequent. This consideration results in a substantial reduction of search space and allows rule discovery in a computationally feasible time. Apriori generates a rule of the form: s =  > (f – s) if and only if the confidence of the rule is above the user-defined threshold. Confidence is essentially the accuracy of the rule and is used in Apriori to rank the rules (Agrawal & Srikant [2]; Mutter et al. [51]).

#### Weighted confidence

The confidence level is used in order to show how often the rule appears to be true. Let Y be the ‘goal’, then the weighted confidence of a rule X → Y can be calculated as the ratio of weighted support of $\left( {X \cup Y} \right)$ over the weighted support of (X).

4 $${\text{Weighted}}\;{\text{Confidence }} = \left( {\frac{{Weighted \;Support \left( {X \cup Y} \right)}}{Weighted \;support \left( X \right)}} \right)$$

For instance, the rule {sex = Male, CA = 3} → {heart disease} has a confidence of 0.2/0.2 = 1.0. It means a patient who is a male and having 3 CA (major vessels coloured by fluoroscopy) has a 100% chance of having heart disease.

### Evaluation

This phase generates rules based on the Apriori algorithm in Weighted Associative Rule Mining. Two sets of rules and confidence scores were generated for the followings:

(i) All features—this includes all the 13 features.
(ii) Selected significant features (8 features).

The following section provides a detailed explanations of the results obtained which are the rules and confidence scores.

## Results (rules and confidence level generated)

The rules and confidence level generated for all the (13) features and the selected significant features (8) are shown in this section.

### All features

Table 8 shows the top 20 rules and confidence scores obtained for all the features using WARM. The rules were sorted by the highest confidence scores.

**Table 8:** Rules generated from all the features using WARM

No	Rules	Confidence
1	Trestbps = unusual Thalach = Tachycardia Exang = No CA = zero Thal = normal = = > class_HD = No Heart Disease	0.96
2	Trestbps = unusual Fbs = FALSE Thalach = Tachycardia Exang = No CA = zero Thal = normal 52 = = > class_HD = No Heart Disease	0.96
3	Sex = Female Exang = No CA = zero = = > class_HD = No Heart Disease	0.96
4	Sex = Female Thalach = Tachycardia Exang = No CA = zero = = > class_HD = No Heart Disease	0.96
5	Sex = Female Exang = No CA = zero Thal = normal = = > class_HD = No Heart Disease	0.96
6	Age = betweenAge Trestbps = unusual Exang = No CA = zero Thal = normal = = > class_HD = No Heart Disease	0.96
7	CP = asymptomatic Slope = flat Thal = reversable = = > class_HD = Heart Disease	0.96
8	Sex = Female Fbs = FALSE Exang = No CA = zero = = > class_HD = No Heart Disease	0.96
9	Sex = Female Fbs = FALSE Thalach = Tachycardia Exang = No CA = zero = = > class_HD = No Heart Disease	0.96
10	Sex = Female Thalach = Tachycardia Exang = No CA = zero Thal = normal = = > class_HD = No Heart Disease	0.96
11	Age = betweenAge Trestbps = unusual Thalach = Tachycardia Exang = No CA = zero Thal = normal 48 = = > class_HD = No Heart Disease	0.96
12	Trestbps = unusual Exang = No CA = zero Thal = normal = = > class_HD = No Heart Disease	0.95
13	Trestbps = unusual Fbs = FALSE Thalach = Tachycardia CA = zero Thal = normal = = > class_HD = No Heart Disease	0.95
14	Age = betweenAge CP = asymptomatic Oldpeak = greaterThanZero Thal = reversable = = > class_HD = Heart Disease	0.95
15	Restecg = normal Thalach = Tachycardia Exang = No CA = zero Thal = normal = = > class_HD = No Heart Disease	0.95
16	CP = asymptomatic Fbs = FALSE Oldpeak = greaterThanZero Thal = reversable = = > class_HD = Heart Disease	0.94
17	Trestbps = unusual Fbs = FALSE Exang = No CA = zero Thal = normal = = > class_HD = No Heart Disease	0.94
18	CP = asymptomatic Exang = Yes Thal = reversable = = > class_HD = Heart Disease	0.94
19	Sex = Male CP = asymptomatic Exang = Yes Oldpeak = greaterThanZero = = > class_HD = Heart Disease	0.94
20	Age = betweenAge CP = asymptomatic Thalach = Tachycardia Oldpeak = greaterThanZero Thal = reversable = = > class_HD = Heart Disease	0.94

The highest confidence level achieved for predicting the risk of having heart disease is 96% and the number of features used to generate this rule is 3(CP, Slope and Thal). This can be clearly seen in Table 8 (Rule Number 7). The rule states that if the value of Chest Pain (CP) is asymptomatic, the slope is flat and the value of Thallium (Thal) is reversible, therefore, the patient has a very high tendency (confidence level = 96%) of having the risk of heart disease. All the highlighted rows in Table 8 show the rules that contributed to the prediction of the risk of having heart disease. Further, the Table 9 is the summary that shows the frequency of each features used in the rules, which were generated from Table 8 (which contains the rules that predicts heart disease). It shows the rule number and the features used in each of the top 20 rules. From the top 20 rules, only 6 rules predicts heart disease and others are non-sick rules which predicts no heart disease.

**Table 9:** Summary of frequency of each features contained in the rules that predicts heart disease (all features)

	Features								
	CP	Slope	Thal	Age	OldPeak	Fbs	Exang	Sex	Thalach
**Rule number**									
7	√	√	√						
14	√			√	√				
16	√		√		√	√			
18	√		√				√		
19	√				√		√	√	
20	√		√	√	√				√
**Total rules**									
6	6	1	4	2	4	1	2	1	1

Although all 13 features have been used for rules and confidence score generation as shown in Table 8, only 9 features have been used for heart disease prediction based on the top 20 rules. The most significant feature in predicting heart disease is CP. This feature exist in all the 6 rules generated that predicts heart disease. Thal and Oldpeak exist in 4 rules out of the 6 rules in predicting heart disease.

### Selected significant features

This section emphasizes on the rules and confidence scores obtained by the selected significant features. Table 10 shows the top 20 rules generated from the significant features using WARM. The confidence score obtained in predicting the risk of having heart disease using 8 selected significant features shows a comparatively high confidence level at 98%. The rule obtained for the top confidence score states as.

**Table 10:** Rules generated from 8 significant features using weighted associative rule mining

No	Rules	Confidence
1	Sex = Female CP = nonAnginalPain Thal = normal = = > class_HD = No Heart Disease	1
2	Sex = Female Exang = No Oldpeak = greaterThanZero CA = zero = = > class_HD = No Heart Disease	1
3	CP = asymptomatic Exang = Yes Oldpeak = greaterThanZero Thal = reversible = = > class_HD = Heart Disease	0.98
4	Sex = Male CP = asymptomatic Exang = Yes Oldpeak = greaterThanZero Thal = reversable = = > class_HD = Heart Disease	0.97
5	CP = asymptomatic Fbs = FALSE Exang = Yes Oldpeak = greaterThanZero Thal = reversable = = > class_HD = Heart Disease	0.97
6	Sex = Female CP = nonAnginalPain = = > class_HD = No Heart Disease	0.97
7	Sex = Female Fbs = FALSE Exang = No Oldpeak = greaterThanZero Thal = normal = = > class_HD = No Heart Disease	0.97
8	Sex = Male CP = asymptomatic CA = one = = > class_HD = Heart Disease	0.97
9	Sex = Female CP = nonAnginalPain Exang = No = = > class_HD = No Heart Disease	0.97
10	CP = asymptomatic Exang = Yes Slope = flat Thal = reversable = = > class_HD = Heart Disease	0.97
11	CP = asymptomatic Exang = Yes Oldpeak = greaterThanZero Slope = flat Thal = reversable = = > class_HD = Heart Disease	0.97
12	Sex = Male CP = asymptomatic Fbs = FALSE Exang = Yes Oldpeak = greaterThanZero Thal = reversable = = > class_HD = Heart Disease	0.97
13	Sex = Female Exang = No CA = zero = = > class_HD = No Heart Disease	0.96
14	Sex = Female Exang = No CA = zero Thal = normal = = > class_HD = No Heart Disease	0.96
15	CP = asymptomatic Slope = flat Thal = reversable = = > class_HD = Heart Disease	0.96
16	Sex = Female Fbs = FALSE Exang = No CA = zero = = > class_HD = No Heart Disease	0.96
17	CP = asymptomatic Oldpeak = greaterThanZero Slope = flat Thal = reversable = = > class_HD = Heart Disease	0.96
18	Sex = Female Fbs = FALSE Exang = No CA = zero Thal = normal = = > class_HD = No Heart Disease	0.96
19	CP = asymptomatic Fbs = FALSE Slope = flat Thal = reversable = = > class_HD = Heart Disease	0.95
20	CP = asymptomatic Fbs = FALSE Oldpeak = greaterThanZero Slope = flat Thal = reversable = = > class_HD = Heart Disease	0.95

**CP = asymptomatic, Exang = Yes, Oldpeak = greaterThanZero, Thal = reversible =  =  > class_HD = Heart Disease.**

which means if Chest Pain (CP) is asymptomatic, exercise-induce angina (Exang) is present, Oldpeak (ST depression induced by exercise relative to rest) is present and, Thallium heart scan (Thal) is reversible then the patient is diagnosed as having heart disease. From the top 20 rules generated, 11 rules are meant for predicting heart disease as highlighted in Table 10. Table 11 shows the summary of the frequency of existence of each features contained in the rules that predicts heart disease. There are a total of 11 rules out of 20 rules generated using significant features to predict the presence of heart disease. The most significant feature that exists in all the positive rules that predicts the Heart Disease is Chest pain (CP). Thallium heart scan (Thal) is seen in 9 out of 11 rules and Oldpeak (ST depression induced by exercise relative to rest) is seen in 7 rules.

**Table 11:** Summary of frequency for each features contained in the rules that predicts heart disease (8 selected features)

	Features							
	CP	Slope	Thal	OldPeak	Fbs	Exang	Sex	CA
**Rule number**								
3	√		√	√		√		
4	√		√	√		√	√	
5	√		√	√	√	√		
8	√						√	√
10	√	√	√			√		
11	√	√	√	√		√		
12	√		√	√	√	√	√	
15	√	√	√					
17	√	√	√	√				
19	√	√			√			
20	√	√	√	√	√			
**Total rules**								
11	11	6	9	7	4	6	3	1

## Discussions

The implementation of WARM on selected significant features managed to achieve the highest confidence score in predicting heart disease which is 98% compared to 96% obtained from all features. It can be concluded that WARM predicts the risk of having heart disease well. From the top 20 rules generated, only 6 rules were based all features. On the other hand, 11 rules from the top 20 generated were based on the selected 8 features.

Studying the top 20 rules generated revealed some significant information. These findings were validated by a cardiologist:-

- Asymptomatic chest pain, positive exercise-induced angina, Oldpeak > 0 and reversible thallium heart scan implies the presence of heart disease.
  CP = asymptomatic, Exang = Yes, Oldpeak = greaterThanZero, Thal = reversible =  =  > class_HD = Heart Disease
- Asymptomatic chest pain is one of the most important features as it appears in all the rules generated in detecting heart disease.
- Reversible thallium heart scan and Oldpeak greater than zero are positively correlated with heart disease.
- Males are more prone to have heart disease compared to females as all the sick rules stated sex as male and the healthy rules stated sex as female.
- There is a strong negative correlation between CA and Thal for heart disease prediction.
- The most common features that exist in healthy rules are Sex = Female, Exang (Exercise induce angina) = No and CA (Number of major vessels coloured by fluoroscopy) = Zero. A patient will be predicted as not having heart disease if the patient is female, angina is not induced by exercise and has no major vessels coloured by fluoroscopy.
- Slope is not featured in any of the healthy rules.
- This study managed to determine the processes involved in obtaining significant features and to devise a scoring mechanism to obtain the strength of each feature. This will enable for the correct weight to be imposed on each of the significant features to be used in WARM for predicting heart disease. The confidence score obtained in this study is the highest obtained in heart disease prediction using WARM based on the UCI dataset. This study can be used as a guide for computing thestrength scores of significant features found in other heart disease datasets.

## Comparative analysis with existing work

**Fig. 3:**
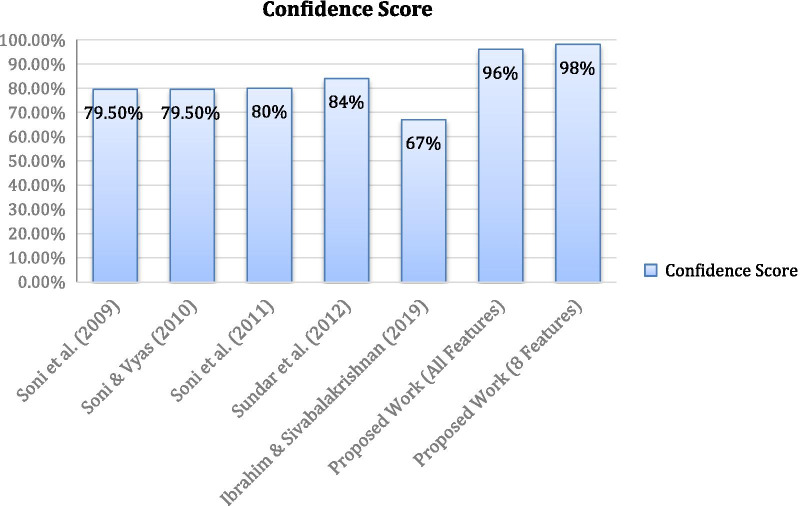
Result comparison on WARM using UCI Cleveland heart disease dataset

This section performs comparison between the proposed work and existing works using WARM. The results obtained in this research proved that the weighted scores imposed on WARM for 8 significant features have the highest confidence score of 98% compared with other existing studies. Figure 3 shows the confidence score of all the existing studies on WARM that used the UCI Cleveland heart disease dataset in comparison with the proposed work. The confidence score obtained by both the experiments which includes all features and significant features in predicting heart disease using WARM achieved a significant difference in terms of the confidence score achieved compared to previous studies. The use of the significant features score in WARM provides the highest confidence of 98% predicting heart disease.

Table 12 presents a comparative analysis of WARM using significant features versus existing results of ARM in heart disease prediction. Rules that gave the highest confidence scores were retrieved and compared in this table. Research by Said et al. [41] and Khare and Gupta [24] showed lower confidence scores compared to this research. Although Sonet et al., [45] managed to obtain a confidence score of 99%, the rule generated for this score is questionable. The rule stated that if a patient has diabetes, then the patient will have heart disease. Although the risk of having heart disease is proven to be higher in diabetic patient, this rule cannot be generalized for all diabetic patients. This is the result of bias that might have existed in their dataset. The dataset used in their study is collected from 4 different medical institutions with a total of 131 records and is not an open dataset. Besides that, the dataset contained different features from the dataset used in this study.

**Table 12:** Comparative Analysis of Weighted Associative analysis and Associative Rule Mining in predicting heart disease

Research	Confidence Score (%)	Rules	No of attributes in highest confidence rule	Technique	Dataset
Nahar et al. [36]	96	Chest_Pain_Type = asympt, Slope = flat, Thal = rev	3	ARM	UCI
Said et al. [41]	91	Chest Pain Type = asymptomic and Thal = reversible defect	2	ARM	UCI
Khare and Gupta [24]	94	Thal = reversible_defect, CP = asymptomatic, Exercise_Induced_Angina = yes	3	ARM	UCI
Sonet et al. [41]	97	Lack-of-Exercise = yes, Stress = yes, BP = high, Smoking = yes, Diabetes = yes ֜	5	ARM	Data collected from 4 medical institutions (131 records)
	99	Diabetes	1	ARM	
Soni and Vyas [48]	79.5	NA	NA	WARM	UCI
Soni et al. [46]	80	NA	NA	WARM	UCI
Sundar et al. [50]	84	NA	NA	WARM	UCI
Ibrahim & Sivabalakrishnan [18]	67	70..79- > yes	1	WARM	UCI
Our Experiment (all features)	96	CP = asymptomatic Slope = flat Thal = reversable	3	WARM	UCI
Our Experiment (8 Significant features)	98	CP = asymptomatic, Exang = Yes, Oldpeak = greaterThanZero, Thal = reversible	4		

This study also benchmarked the rules generated using the UCI dataset by past researches with the rules generated in our study. The extracted healthy rules are shown in Table 13 and sick rules are shown in Table 14. Table 13 shows that our experiment with 8 significant features obtained the optimum confidence score of 100% for predicting healthy rules. The rules retrieved for this stated that if the sex is female, chest paint is non-angina and thallium heart scan is normal, this person is then predicted not to have heart disease.

**Table 13:** Healthy rules extractions

Research	Rules	Confidence Scores
Nahar et al. [36]	Sex = female, Exercise_induced_angina = fal, Number_of_vessels_colored = 0, Thal = nom	98
Said et al. [41]	Sex = female and Exercise_induced_angina = No and Thal = normal	89
Khare et al. [24]	Ca = 0, Thal = normal, Exercise_induced_angina = no	90
Proposed work (with all features)	Trestbps = unusual, Thalach = Tachycardia, Exang = No, CA = zero Thal = normal	96
Proposed Work (with significant features)	Sex = Female, CP = nonAnginalPain, Thal = normal	100

**Table 14:** Sick rules extractions

Research	Rules	Confidence Scores
Nahar et al. [36]	Chest_pain_type = asympt, Slope = flat, Thal = rev	96
Said et al. [41]	Chest pain type = asymptomic and Thal = reversible defect	91
Khare et al. [24]	Thal = reversible_defect, CP = asymptomatic, Exercise_induced_angina = yes	94
Ibrahim and Sivabalakrishnan [19]	70..79- > yes	67
Proposed Work (all features)	CP = asymptomatic, Slope = flat, Thal = reversable	96
Proposed Work (8 significant features)	CP = asymptomatic Exang = Yes Oldpeak = greaterThanZero Thal = reversible	98

Table 14 shows the sick rules together with the highest confidence scores of this research in comparison with other resesarch on associative and WARM for heart disease prediction. This study achieved a confidence score of 98% which is better than all the other predicted sick rules. To the best of our knowledge, the significant features’ weighted scores in our study managed to beat the scores obtained by all other research using ARM and WARM to predict heart disease.

## Conclusion

This research contributed to obtaining the highest confidence score using significant features in WARM for heart disease prediction. Assigning appropriate weight scores have proven to improve the performance of confidence level in the prediction. A set of significant features with different weights to represent the strength of each of the features was used in heart disease prediction. To the best of our knowledge, this is the first study that made use of significant features in executing WARM. This research has also contributed to listing the top rules in predicting heart disease based on the UCI dataset. This is the first research that benchmarked the healthy rules and sick rules with the highest confidence scores. Future researches may look into predicting the risk levels of heart disease, as this will help medical practitioners and patients to gauge their heart disease severity. The algorithm used in this study for measuring weight can be further explored for use with other datasets to cater to other prediction models using the weighted approach. The machine learning techniques used in feature selection phase of this research is limited to the most popular techniques used in heart disease prediction research. Future researchers should look into exploring other machine learning techniques in selecting the significant features.

## Data Availability

The datasets analysed during the current study are available as Cleveland Dataset in UCI Machine Learning Repository, [https://www.kaggle.com/ronitf/heart-disease-uci]

## References

[1] Agarwal R, Mittal M. Inventory classification using multi-level association rule mining. Int J Dec Supp Syst Technol. (IJDSST), 2019;11(2):1–12.

[2] Agrawal R, Srikant R. Fast algorithms for mining association rules. In: Proceedings of 20th international conference very large data bases, VLDB. Vol. 1215, pp. 487–499; 1994.

[3] Akbaş KE, Kivrak M, Arslan AK, Çolak C. Assessment of association rules based on certainty factor: an application on heart data set, in 2019 International artificial intelligence and data processing symposium (IDAP) (pp. 1–5). IEEE; 2019.

[4] Altaf W, Shahbaz M, Guergachi A (2017). Applications of association rule mining in health informatics: a survey. Artif Intell Rev.

[5] Alwidian J, Hammo BH, Obeid N (2018). WCBA: weighted classification based on association rules algorithm for breast cancer disease. Appl Soft Comput.

[6] American Heart Association. Heart disease and stroke statistics 2017 at-a-glance. *Geraadpleegd van:* https://healthmetrics.heart.org/wp-content/uploads/2017/06/Heart-Disease-and-Stroke-Statistics-2017-ucm_491265.pdf.

[7] Amin MS. Identifying significant features and data mining techniques in predicting cardiovascular disease; 2018.

[8] Amin MS, Chiam YK, Varathan KD (2019). Identification of significant features and data mining techniques in predicting heart disease. Telematics Inform.

[9] Bashir, S., Khan, Z. S., Khan, F. H., Anjum, A., & Bashir, K. (2019). Improving heart disease prediction using feature selection approaches. In *2019 16th International Bhurban Conference on Applied Sciences and Technology (IBCAST)* (pp. 619–623). IEEE.

[10] Cengiz AB, Birant KU, Birant D. Analysis of pre-weighted and post-weighted association rule mining, in 2019 Innovations in Intelligent Systems and Applications Conference (ASYU) (pp. 1–5). IEEE.

[11] Chauhan A, Jain A, Sharma P, Deep V. Heart disease prediction using evolutionary rule learning, in 2018 4th International conference on computational intelligence & communication technology (CICT) (pp. 1–4). IEEE; 2018.

[12] Dey L, Mukhopadhyay A (2019). Biclustering-based association rule mining approach for predicting cancer-associated protein interactions. IET Syst Biol.

[13] Dua, D., Graff, C. UCI machine learning repository [http://archive.ics.uci.edu/ml]. Irvine, CA: University of California, School of Information and Computer Science; 2019.

[14] Domadiya N, Rao UP (2018). Privacy-preserving association rule mining for horizontally partitioned healthcare data: a case study on the heart diseases. Sādhanā.

[15] Domadiya N, Rao UP (2019). Privacy preserving distributed association rule mining approach on vertically partitioned healthcare data. Procedia Comput Sci.

[16] Fitriyani NL, Syafrudin M, Alfian G, Rhee J (2020). HDPM: an effective heart disease prediction model for a clinical decision support system. IEEE Access.

[17] Han J, Pei J, Kamber M (2011). Data mining: concepts and techniques.

[18] Ibrahim SP, Sivabalakrishnan M. An enhanced weighted associative classification algorithm without preassigned weight based on ranking hubs. Int J Adv Comput Sci Appl. 10(10); 2019.

[19] Ibrahim SS, Sivabalakrishnan M. An evolutionary memetic weighted associative classification algorithm for heart disease prediction. In Recent Advances on Memetic Algorithms and its Applications in Image Processing (pp. 183–199). Springer, Singapore; 2020.

[20] James SL, et al. Global, regional, and national incidence, prevalence, and yearslived with disability for 354 diseases and injuries for 195 countries and territories, 1990–2017: a systematic analysis for the Global Burden of Disease Study 2017. Lancet392 (10159), 1789–1858; 2018. 10.1016/S0140-6736(18)32279-7 PMC6227754 30496104

[21] Jabbar MA, Deekshatulu BL, Chandra P. Graph based approach for heart disease prediction. In Proceedings of the third international conference on trends in information, telecommunication and computing. New York, NY: Springer. 2013. p. 465–474.

[22] Kannan AG, Castro TARVC, BalaSubramanian R. A comprehensive study on various association rule mining techniques; 2018.

[23] Khan SA, Yadav SK. Class-based associative classification using super subsets to predict the by-diseases in thyroid disorders. in International conference on advances in computational intelligence and informatics (pp. 301–308). Springer, Singapore; 2019.

[24] Khare S, Gupta D. Association rule analysis in cardiovascular disease. In: Cognitive Computing and Information Processing (CCIP), 2016 Second International Conference on (pp. 1–6). IEEE; 2016.

[25] Kharya S, Soni S, Swarnkar T. Weighted Bayesian association rule mining algorithm to construct Bayesian Belief network. In: 2019 International conference on applied machine learning (ICAML) (pp. 27–33). IEEE.

[26] Lakshmi KS, Vadivu G. A novel approach for disease comorbidity prediction using weighted association rule mining. *Journal of Ambient Intelligence and Humanized Computing*, 1–8; 2019.

[27] Lakshmi KP, Reddy CRK. Fast rule-based heart disease prediction using associative classification mining, in 2015 International conference on computer, communication and control (IC4) (pp. 1–5). IEEE; 2015.

[28] Mahdi MA, Al-Janabi S. A novel software to improve healthcare base on predictive analytics and mobile services for cloud data centers, in International conference on big data and networks technologies (pp. 320–339). Springer, Cham; 2019.

[29] Maji S, Arora S. Decision tree algorithms for prediction of heart disease. In Information and communication technology for competitive strategies (pp. 447–454). Springer, Singapore; 2019.

[30] Malarvizhi SP, Sathiyabhama B (2016). Frequent pagesets from web log by enhanced weighted association rule mining. Clust Comput.

[31] Methaila A, Kansal P, Arya H, Kumar P. Early heart disease prediction using data mining techniques. Comput Sci Inf Technol J. 53–59; 2014.

[32] Mohammed KI, Zaidan AA, Zaidan BB, Albahri OS, Albahri AS, Alsalem MA, Mohsin AH (2020). Novel technique for reorganisation of opinion order to interval levels for solving several instances representing prioritisation in patients with multiple chronic diseases. Comput Methods Programs Biomed.

[33] Mohammed KI, Jaafar J, Zaidan AA, Albahri OS, Zaidan BB, Abdulkareem KH, Alamoodi AH (2020). A uniform intelligent prioritisation for solving diverse and big data generated from multiple chronic diseases patients based on hybrid decision-making and voting method. IEEE Access.

[34] Murphy SL, Xu J, Kochanek KD, Arias E. Mortality in the United States, 2017. NCHS data brief, no 328. Hyattsville, MD: National Center for Health Statistics; 2018. 30500322

[35] Mutter S, Hall M, Frank E. Using classification to evaluate the output of confidence-based association rule mining, in AI 2004: Advances in, Artificial Intelligence, 133–148; 2005.

[36] Nahar J, Imam T, Tickle KS, Chen YPP. Association rule mining to detect factors which contribute to heart disease in males and females. Expert Syst Appl. 2013;40(4):1086–1093.

[37] Nguyen T (2015). Classification of healthcare data using genetic fuzzy logic system and wavelets. Expert Syst Appl.

[38] Orphanou K, Dagliati A, Sacchi L, Stassopoulou A, Keravnou E, Bellazzi R (2018). Incorporating repeating temporal association rules in naïve bayes classifiers for coronary heart disease diagnosis. J Biomed Inform.

[39] Park HY, Lim DJ (2021). A design failure pre-alarming system using score-and vote-based associative classification. Expert Syst Appl.

[40] Roth GA (2018). Global, regional, and national age-sex-specific mortality for 282 causes of death in 195 countries and territories, 1980–2017: a systematic analysisfor the Global Burden of Disease Study 2017. Lancet.

[41] Said IU, Adam AH, Garko AB. Association rule mining on medical data to predict heart disease. Int J Sci Technol Manage. 2015. 26–35.

[42] Shuriyaa B, Rajendranb A (2018). Cardio vascular disease diagnosis using data mining techniques and ANFIS approach. Int J Appl Eng Res.

[43] Sim DYY, Teh CS, Ismail AI (2017). Improved boosting algorithms by pre-pruning and associative rule mining on decision trees for predicting obstructive sleep apnea. Adv Sci Lett.

[44] Singh J, Kamra A, Singh H. Prediction of heart diseases using associative classification, in 2016 5th International conference on wireless networks and embedded systems (WECON) (pp. 1–7). IEEE; 2016.

[45] Sonet, K. M. H., Rahman, M. M., Mazumder, P., Reza, A., & Rahman, R. M. (2017). Analyzing patterns of numerously occurring heart diseases using association rule mining. In *2017 Twelfth International Conference on Digital Information Management (ICDIM)* (pp. 38–45). IEEE.

[46] Soni J, Ansari U, Sharma D, Soni S (2011). Intelligent and effective heart disease prediction system using weighted associative classifiers. International Journal on Computer Science and Engineering.

[47] Soni S, Pillai J, Vyas OP. An associative classifier using weighted association rule. In: 2009 World Congress on Nature & Biologically Inspired Computing (NaBIC). IEEE. 2009. p. 1492–1496.

[48] Soni S, Vyas OP (2010). Using associative classifiers for predictive analysis in health care data mining. Int J Comput Appl.

[49] Srinivas K, Reddy BR, Rani BK, Mogili R. Hybrid Approach for prediction of cardiovascular disease using class association rules and MLP. Int J Electr Comput Eng. (2088–8708), 6(4); 2016.

[50] Sundar NA, Latha PP, Chandra MR (2012). Performance analysis of classification data mining techniques over heart disease database. Int J Eng Sci Adv Technol.

[51] Taihua W, Fan G. Associating IDS alerts by an improved apriori algorithm. in Third international symposium on intelligent information technology and security informatics, 2010 Jinggangshan, China (pp. 478–482). IEEE; 2010.

[52] Thanigaivel R, Kumar KR (2016). Boosted apriori: an effective data mining association rules for heart disease prediction system. Middle-East J Sci Res.

[53] UCI Machine Learning Repository: Heart Disease Data Set; 2010. http://archive.ics.uci.edu/ml/datasets/Heart+Disease

[54] Vasanthanageswari S, Vanitha M (2018). Predicting risk factor of congenital heart defect using association rule mining technique. Int J Pure Appl Math.

[55] Wei-Jia L, Liang M, Hao C (2017). Particle swarm optimisation-support vector machine optimised by association rules for detecting factors inducing heart diseases. J Intell Syst.

[56] World Health Organization. Global action plan for the prevention and control of non-communicable diseases 214–2020. ISBN 978 92 4 150623 6. Geneva 2013; 2013.

